# Introduction to the research topic meaning in mind: semantic richness effects in language processing

**DOI:** 10.3389/fnhum.2013.00723

**Published:** 2013-11-05

**Authors:** Penny M. Pexman, Paul D. Siakaluk, Melvin J. Yap

**Affiliations:** ^1^Department of Psychology, University of CalgaryCalgary, AB, Canada; ^2^Department of Psychology, University of Northern British ColumbiaPrince George, BC, Canada; ^3^Department of Psychology, National University of SingaporeSingapore, Singapore

**Keywords:** semantic processing, word meaning, semantic richness, embodied cognition, concrete concepts, abstract concepts, lexical processing

The ultimate goal of reading is to extract meaning from printed words. However, the mechanisms that mediate orthography and semantics are not well-understood, and have rarely been implemented in computational models. To address this puzzle, one of the strategies cognitive scientists have begun to use is to examine semantic richness effects. Semantic richness effects refer to the finding that words associated with relatively more semantic information are recognized faster and more accurately, due to their possessing richer, better-specified semantic representations. Importantly, semantic richness is not a unitary concept. Instead, it draws on various theoretical perspectives and can vary along multiple dimensions. Thus, by examining which dimensions of semantic richness influence visual word recognition behavior, we gain insight about which theoretical perspectives seem to be promising descriptions of the process by which meaning is extracted from print. Our goal for this Frontiers Research Topic was to highlight the latest findings regarding semantic richness and theoretical developments on the issue of semantic processing. Our hope was to provide a forum for state-of-the-art research in this field, and to foster new theoretical advances. The 17 contributions that comprise the Research Topic certainly represent the state of the art; methodologies include ERP, fMRI, TMS, and behavioral approaches, and involve both intact and patient populations. Together, these contributions give rise to a number of inferences about semantic richness effects and implications of those effects for our understanding of semantic processing effects in visual word recognition.

## Meaning is multidimensional

The Research Topic contributions build on previous literature, providing further empirical support for several semantic richness dimensions and the frameworks from which those dimensions are derived. Gould et al. (2012); Recchia and Jones (2012); Yap et al. (2012) report semantic neighborhood effects (faster responses for words with more semantic neighbors or denser semantic neighborhoods) in naming and lexical decision tasks, providing evidence that lexical co-occurrence is an important dimension in semantic memory. Hargreaves and Pexman (2012); Taler et al. (2013) show that lexical decision performance is facilitated for words with more meaning senses, providing support for the notion that meaning information is represented in a distributed fashion. The typicality effects reported by Woollams (2012) support the claim that words' feature structure is important to semantic memory. Further, Recchia and Jones (2012); Yap et al. (2012) show that words that generate more features in feature listing tasks produce faster naming, lexical decision, and semantic categorization responses, Hargreaves et al. (2012a) report that those words are also better remembered in free recall. Finally, there is evidence supporting embodied frameworks of semantic memory from studies reported by Esopenko et al. (2012); McNorgan (2012). Further support for the embodied framework is provided by Hansen et al. (2012); Hargreaves et al. (2012b); Newcombe et al. (2012); Tousignant and Pexman (2012); Yap et al. (2012), as all of these studies report body-object interaction effects (faster processing for words that refer to objects the human body can easily interact with) in tasks that include naming, lexical decision, and semantic categorization. Convergent evidence that perceptual and sensorimotor information are important dimensions of meaning comes from the observations of Hargreaves and Pexman (2012); Newcombe et al. (2012); Yap et al. (2012) by which imageability effects (faster responses for words that are associated with imagery) are reported in a number of word recognition tasks.

In addition, in the contributions of Hargreaves and Pexman (2012); Newcombe et al. (2012); Recchia and Jones (2012); Yap et al. (2012) there are demonstrations that multiple semantic richness effects can be observed simultaneously, suggesting that each richness dimension explains unique variance in word recognition behavior. The implication is that no single dimension (and associated framework) will be sufficient to explain the process by which meaning is derived from print. Instead, as argued by Dilkina and Lambon Ralph (2013); Jones and Golonka (2012); Kalénine et al. (2012), semantic memory is multidimensional.

## Semantic processing is variable and dynamic

The findings of Kalénine et al. (2012); Woollams (2012) support the inference that semantic processing is variable as a function of disease. By studying the dimensions of meaning that are more resistant to brain damage these studies provide important new clues about the structure of meaning in the mind. The contributions of Hargreaves and Pexman (2012); Hansen et al. (2012) show that semantic processing is variable as a function of both short-term and long-term experience. Further variability is revealed in Jones and Golonka (2012); Kalénine et al. (2012); Rabovsky et al. (2012); Taler et al. (2013), where the timecourse of processing is examined in order to dissociate richness dimensions. Results show, first, that semantic information is generated quite early in the process of word recognition and, second, that different dimensions of meaning may be influential at different times as semantic processing unfolds.

Contributions by Gould et al. (2012); Hansen et al. (2012); Hargreaves and Pexman (2012); Recchia and Jones (2012); Tousignant and Pexman (2012); Yap et al. (2012) demonstrate that the process of generating meaning from print is a dynamic one, where contextual factors like task demands shape the information that is generated from letter strings. These demonstrations are consistent with the notion of a flexible lexical processor (Balota and Yap, 2006) that is sensitive to task contexts so as to optimize task performance via attentional control. The present findings also permit the inference that the semantic richness effects observed in a given task do not provide veridical insight about static semantic representations. Semantic representation is not fixed and so cannot be revealed in a single task or context (Kiefer and Pulvermüller, 2012). Rather, meaning is actively constructed and shaped to meet task demands. Dimensions that are important in one context may not be important in others. Certainly, it now seems clear that there are many candidate dimensions of meaning, but the context will dictate the actual effects observed.

## Future directions: abstract meaning and other challenges

As has been typical in the lexical semantic literature, most of the contributions in this Research Topic focus on semantic processing of concrete words, like TRUCK, where the word refers to an object or entity in the world. As such, while we know quite a lot about how concrete meanings might be processed, we know much less about how abstract meanings are understood. This is problematic because abstract words make up a large part of the average person's vocabulary; the focus on concrete word meaning creates a situation where we are studying only part of the human lexicon. In two of the present papers, however, the authors use semantic richness effects to begin to study semantic processing of abstract words, like TRUTH. Newcombe et al. (2012); Recchia and Jones (2012) explore semantic richness dimensions that could be relevant to abstract word meaning. Since many of the richness dimensions that are influential for concrete words are not as relevant to the meanings of abstract words (e.g., those dimensions that refer to objects), the richness dimensions that influence abstract word meaning are somewhat different. For instance, Newcombe et al. (2012) show that while body-object interaction is an important dimension for concrete words, emotion information is important for abstract words, consistent with predictions derived from the embodied cognition framework of Kousta et al. (2010). In addition, Recchia and Jones (2012) show that richer linguistic contexts (larger semantic neighborhoods) facilitate abstract word processing. These contributions are first steps in the study of abstract word meaning, and this issue will need to be taken up in future research.

We suggest, further, that future research on this topic should continue to explore several of the other important avenues opened here, for instance, the role of individual differences in semantic processing and the joint effects of different semantic richness dimensions. There are additional issues that have not yet received much attention but will be important; for instance, the issue of whether semantic richness dimensions influence processing in a linear or non-linear manner, and the extent to which richness effects extend beyond single-word contexts to influence processing of phrases and sentences. These and other research questions should be addressed in order that we are able to further refine our understanding of how word meaning is processed in mind and brain.

## Conclusion

The contributions in this Frontiers Research Topic highlight a number of dimensions of semantic richness and the contexts in which they are observed. The contributions cohere around several insights: multiple types of information are constitutive of word meaning, and semantic processing is a dynamic process that must be tracked with careful consideration of context and other sources of variability; the challenges for theories of semantic meaning are to capture this multidimensionality, and to extend their reach to include abstract meanings.
